# Bioactive Potential and Chemical Composition of Coffee By-Products: From Pulp to Silverskin

**DOI:** 10.3390/foods12122354

**Published:** 2023-06-13

**Authors:** Marlene Machado, Liliana Espírito Santo, Susana Machado, Joana C. Lobo, Anabela S. G. Costa, Maria Beatriz P. P. Oliveira, Helena Ferreira, Rita C. Alves

**Affiliations:** 1 Network of Chemistry and Technology/Associated Laboratory for Green Chemistry (REQUIMTE/LAQV), Laboratory of Bromatology and Hydrology, Department of Chemical Sciences, Faculty of Pharmacy, University of Porto, 4050-313 Porto, Portugal; 2 Network of Chemistry and Technology/Unit on Applied Molecular Biosciences (REQUIMTE/UCIBIO/i4HB), Laboratory of Microbiology, Department of Biological Sciences, Faculty of Pharmacy, University of Porto, 4050-313 Porto, Portugal

**Keywords:** nutritional composition, antioxidant activity, chlorogenic acids

## Abstract

Processing the coffee cherry into roasted beans generates a large amount of by-products, which can negatively impact the environment. The aim of this study was to analyze the bioactive potential and chemical composition of different coffee by-products (pulp, husk, parchment, silverskin, defective beans, and green coffee sieving residue) having in mind their bioactive potential for health and well-being. The coffee by-products showed a distinct nutritional composition. The content of ash, protein, fat, and total dietary fiber was significantly higher (*p* < 0.05) in coffee pulp (10.72% dw), silverskin (16.31% dw), defective beans (8.47% dw), and parchment (94.19% dw), respectively. Defective beans and the sieve residue exhibited a higher content of total phenolics (6.54 and 5.11 g chlorogenic acid eq./100 g dw, respectively) as well as higher DPPH^•^ scavenging activity (3.11 and 2.85 g Trolox eq./100 g, respectively) and ferric-reducing antioxidant power (17.68 and 17.56 g ferrous sulfate eq./100 g dw, respectively). All the coffee by-products considered in this study are sources of caffeine and chlorogenic acids, in particular 5-caffeoylquinic acid (5.36–3787.58 mg/100 g dw, for parchment and defective beans, respectively). Thus, they can be recycled as functional ingredients for food, cosmetic and/or pharmaceutical industries, contributing to the social, economic, and environmental sustainability of the coffee industry.

## 1. Introduction

Coffee is one of the world’s most popular beverages due to the organoleptic and stimulating properties of its beans [1]. Coffee beverages represent 75% of the non-alcoholic beverage market and their consumption is increasing [2]. In 2020, around 10.52 million tonnes of green coffee beans were produced, with Brazil, Vietnam, and Colombia being the main producers [3]. As a consequence of this large market, the coffee industry is responsible for generating large amounts of by-products including pulp, husk, parchment, defective coffee beans, green coffee sieving residue, and silverskin [1].

The pulp, husk, and parchment result from cherry pulping and dehulling, and have been exploited as biofuels, biosorbents, feed, enzyme sources, and infusions [4,5]. Defective coffee beans (immature, broken, and damaged by insects) represent about 15–20% of the total coffee production and are usually marketed in the country of origin as low-quality coffee [6]. Before roasting, green coffee beans still need to go through a sieving process to ensure a uniform bean size and remove residual non-bean particles. Indeed, coffee beans of different sizes can reduce roasting quality, since the smaller ones can be over-roasted and the larger ones under-roasted, affecting the beverage’s flavor and aroma [7]. This sieving residue is a waste scarcely described in the literature, which consists of green coffee beans/particles of different sizes and residues of parchment and raw silverskin (which is residually detached due to the beans impact with each other during sieving). This by-product is usually produced in roasting industries and discarded, with no valorization known to date. Coffee silverskin in particular (the thin tegument that covers the raw coffee beans) is mainly released in the subsequent step, i.e., during the beans roasting, due to their expansion along the thermal procedure. Although there are already some studies investigating the incorporation of silverskin in food products [8,9], that is still not a reality, and firelighter production remains its major application [10]. 

In general, the great majority of coffee by-products are still discarded within the environment in high amounts, which can contaminate soil and water [11], particularly due to their high organic load and the presence of caffeine and phenolic compounds that can affect soil microbiota and ecosystems. Furthermore, it has been shown that chlorogenic acid (CGA) and caffeine have phytotoxic properties, with a negative impact on seed germination and plant growth [12,13]. Therefore, strategies to valorize these by-products are needed to improve environmental, economic, and social sustainability of coffee industry [14]. 

Besides caffeine and phenolics, which have known health beneficial properties [15,16], coffee by-products are also rich in carbohydrates, pectins, proteins, vitamins and other bioactive components and nutrients, which can be exploited in a food security context [5]. Notably, coffee pulp/husk has been approved as a novel food under the EU Regulation (EU) 2015/2283 [17]. 

In turn, consumers are increasingly opting for plant-based diets to decrease their environmental footprint and improve their health. In fact, the consumption of plant-based foods has been associated with a lower incidence of diseases such as diabetes, cancer, or cardiovascular disease [18]. Exploring coffee by-products as new sources of nutrients and bioactive compounds can be an effective strategy aligned with circular economy principles [18]. Some studies have incorporated different coffee by-products or derived ingredients into food products (such as muffins, cakes, and bread), with good acceptability [19,20,21]. Furthermore, informing the consumer about the presence of an agri-food by-product in a food product seems to increase its acceptability [22]. Moreover, coffee by-products also have potential for the dermocosmetic industry, due to their richness in CGA and caffeine. Indeed, it has been demonstrated that CGA can scavenge DPPH^•^ and ABTS^•+^ radicals and increase collagen synthesis in human dermal fibroblasts. In turn, caffeine can increase the efficacy of sunscreens, and also boost hair growth by improving microcirculation and inhibiting 5-α-reductase [23].

In a previous study presented at “The 3rd International Electronic Conference on Foods: Food, Microbiome, and Health—A Celebration of the 10th Anniversary of Foods’ Impact on Our Wellbeing” [24], the CGA profiles of two coffee by-products (pulp and silverskin) were studied and compared with green and roasted coffee beans. The results showed that although coffee pulp has a higher CGA content compared to silverskin, these results were lower than green and roasted coffee beans. However, the amount of CGA found in coffee by-products was significant, and its bioactivity should be further explored. Pulp [25,26,27], husk [4,28,29,30,31], silverskin [1,32,33,34,35], and defective coffee beans [6,36,37,38] have already been well characterized in terms of their nutritional composition and phenolic profile, and with regard to various factors such as species, geographical origin, and type of processing. In comparison, there are only a few studies on parchment in this field [39,40,41,42]. Research on parchment is indeed more focused on coffee production and processing, as well as biofuel production. Despite the fact that the above-mentioned by-products have already been characterized, the wide variety of analysis methodologies and expression of results makes it challenging to compare coffee by-products. 

Thus, the present study aimed to ascertain the chemical and bioactive composition of various by-products discarded during coffee processing, from the field to the roast, i.e., pulp, husk, parchment, defective beans, sieving residue, and silverskin; to corroborate previous results; and to elucidate the main differences between coffee by-products to better determine their potential applications. To our knowledge, the residue from sieving green coffee beans has not previously been chemically characterized. 

## 2. Materials and Methods

### 2.1. Reagents and Standards

Kjeldahl tablets (without addition of Se and Hg) were obtained from Merck (Darmstadt, Germany). The standards of chlorogenic acids (5-, 4-, and 3-caffeoylquinic acids), caffeine, 5-hydroxymethylfurfural, Folin–Ciocalteu reagent, sodium nitrite, epicatechin, ferrous sulfate heptahydrate, and trolox, as well as 2,2 diphenyl-1-picrylhydrazyl radical (DPPH^•^), ferric chloride, aluminum chloride, 2,4,6 tripyridyl-S-triazine (TPTZ), sodium acetate, and total dietary fiber assay kit were all obtained from Sigma-Aldrich (St. Louis, MO, USA). Absolute ethanol (≥99.8%) and HPLC-grade methanol were obtained from Honeywell Riedel-de Haën (Seelze, Germany). Glacial acetic acid was from VWR Chemicals (Fontenay-sou-Bois, France). A Seralpur PRO 60 CN and Seradest LFM 20 water purification system was used to obtain ultrapure water. All other chemicals were of analytical grade from several suppliers: Panreac (Barcelona, Spain), Merck (Darmstadt, Germany), and Carlo Erba (Emmendingen, Germany).

### 2.2. Samples and Sample Preparation

Dried pulp from the wet method processing (*Coffee arabica*, Colombia), husks from the dry method processing (*Coffee canephora*, Panamá), and green coffee sieving residue were kindly provided by a coffee importer and roasting company JMV—José Maria Vieira, S.A. (Portugal). Parchment (*Coffee arabica*, Portugal) were provided directly by a national coffee producer (Quinta do Avô João, São Miguel, Azores, Portugal). Silverskin and defective coffee beans (broken and immature) were provided by a Portuguese coffee roasting company (BICAFÉ—Torrefação e Comércio de Café, Lda). Silverskin, defective beans, and the sieving residue consisted of a mixture of two coffee species (*C. arabica* and *C. canephora*, approximately 40%:60%, respectively, obtained from commercial coffee blends of those coffee industries, and representative of their main wastes). After reception, the samples were ground (Grindomix GM 200, Retsch, Haan, Germany), homogenized, and sieved through a sieve with a mesh diameter of 1 mm.

### 2.3. Nutritional Analysis

Total nitrogen was estimated by Kjeldahl analysis (AOAC 984.13) [43]. The protein content was then calculated by multiplying the total nitrogen with the conversion factors 5.6 (pulp and husk) [44], 5.33 (parchment and silverskin) [45], and 5.24 (defective beans and sieving residue) [46]. Ashes were determined by the incineration of samples in a muffle furnace (Thermolyne Muffle Furnace, model 48000, Suwanee, GA, USA) at 575 °C for 72 h (until it turned white) [47]. Insoluble and total fiber content was analyzed through an enzymatic-gravimetric method using the total dietary fiber assay kit (AOAC 985.29 and 991.43, respectively). Total lipids were determined by the Soxhlet method (AOAC 991.36) [43]. Available carbohydrates and soluble fiber were calculated by difference. The determinations were carried out in triplicate. 

### 2.4. Hydroethanolic Extracts

Briefly, 0.4 g (defective beans and sieving residue) or 1.2 g (pulp, husk, parchment and silverskin) and 40 mL of a hydroethanolic solvent (1:1) were mixed under shaking in a Multi Reax (2000 rpm, 30 min, Heidolph, Schwabach, Germany) at room temperature. After centrifugation (5000 rpm, 5 min, Megafuge 16 centrifuge, Heraeus, Hanau, Germany) the supernatants were collected and stored at –20 °C prior to analysis. All extracts were prepared in triplicate. 

### 2.5. Estimation of Antioxidant Compounds

#### 2.5.1. Total Phenolic Content (TPC)

TPC of extracts was determined spectrophotometrically according to Alves et al. [48] with minor modifications. Briefly, 30 μL of extract (in case of parchment) or diluted extract (1:10) were mixed with 150 μL of the Folin–Ciocalteu reagent (1:10) and 120 μL of a Na_2_CO_3_ (7.5% *m*/*v*). The mixture was incubated for 15 min in the dark at 45 °C to allow its reaction. After 30 min at room temperature, the absorbance of the resulting blue color solution was measured at 765 nm (BioTek Instruments, Inc., Winooski, VT, USA). The calibration curve with chlorogenic acid (20–160 mg/L, *r* = 0.9989) was prepared to obtain a correlation between sample absorbance and standard concentrations. TPC was expressed as g of chlorogenic acid equivalents (CGAE)/100 g of dry weight. All measurements were performed in triplicate.

#### 2.5.2. Total Flavonoids Content (TFC)

TFC was determined, in triplicate, by a colorimetric assay based on the formation of a flavonoid-aluminum complex according to Costa et al. [34] with minor modifications. Briefly, 30 μL of extract (in case of parchment) or diluted extract (1:10) were mixed with 75 μL of distilled water and 45 μL of sodium nitrite (1%). After 5 min of incubation at room temperature, 45 μL 5% AlCl_3_ were added. After waiting 1 min, 60 μL of 1 M NaOH and 45 μL of ultrapure water were added. Absorbance measurements were performed at 510 nm using a Synergy HT Microplate Reader. Catechin was used as reference to prepare a standard calibration curve (2.5–200 μg/mL, *r* = 0.9998). TFC was expressed as g of catechin equivalents (CE)/100 g of dry weight. 

### 2.6. In Vitro Antioxidant Activity

#### 2.6.1. DPPH^•^ Scavenging Activity

The DPPH^•^ radical dot inhibition of the extracts was evaluated according to Costa et al. [34] with minor modifications. Briefly, 30 μL of extract (in case of parchment) or diluted extract (1:10) were mixed with an ethanolic solution (270 μL) of DPPH^•^ (6 × 10^−5^ mol/L). The absorbance was measured at 525 nm every 2 min for 40 min to observe the reaction kinetics (endpoint at 40 min). A calibration curve was prepared using trolox as standard in the range of 5.62 to 75.87 mg/L (*r* = 0.9896). The results were expressed as g of trolox equivalent (TE)/100 g of dry weight. Measurements were conducted in triplicate.

#### 2.6.2. Ferric Reducing Antioxidant Power (FRAP)

FRAP assay was carried out according to Benzie and Strain [49] with minor modifications of Alves et al. [48]. Briefly, 30 μL of extract (in case of parchment) or diluted extract (1:50) were added to 265 μL of FRAP reagent. The mixture was incubated at 37 °C in the dark for 30 min. The activity for reducing iron (Fe^3+^) to the ferrous form (Fe^2+^) of the extract was determined at 595 nm in a Synergy HT Microplate Reader. The results were compared with a standard curve of ferrous sulfate in the range of 50 to 600 μmol/L (*r* = 0.9997). All measurements were performed in triplicate, and results were expressed as g of ferrous sulphate equivalents (FSE)/100 of dry weight.

### 2.7. Caffeine, Caffeoylquinic Acids, and 5-Hydroxymethylfurfural 

The hydroethanolic extracts prepared as described in Section 2.4. were filtered through a 0.45 μm nylon syringe filter and analyzed by HPLC-DAD. The chromatographic analysis was performed in a HPLC integrated system from Jasco (Jasco, Tokyo, Japan. This system consisted of an LC-Net II/ADC hardware interface, an automatic sampler (Jasco AS-2057 Plus; Jasco, Tokyo, Japan), a pump (Jasco PU-2089 Plus; Jasco, Tokyo, Japan), a multi-wavelength diode-array detector (DAD, Jasco MD-2018 Plus; Jasco, Tokyo, Japan), and a column oven (Jasco CO-2060 Plus; Jasco, Tokyo, Japan). The gradient elution used was the following: 0 min, 5% B; 40 min, 25% B; 55 min, 45% B; 60 min, 60% B; 65 min, 5% B (solvent A: 0.5% acetic acid; solvent B: 100% methanol), with a flow rate of 1.1 mL/min. The chromatographic column was a Zorbax-SB-C18 (5 μm, 250 mm × 4.6; Agilent Technologies, Santa Clara, CA, USA), at 28 °C. The caffeine, 5-hydroxymethylfurfural, and caffeoylquinic acids were monitored at 274, 280, and 320 nm, respectively. The compound identifications were performed by comparing retention times and co-elution with authentic standards, and by UV absorption spectral analysis. Caffeine, caffeoylquinic acids (3-, 4-, 5-CQA), and 5-hydroxymethylfurfural (HMF) contents were expressed as mg/100 g dw. All determinations were performed in triplicate. 

### 2.8. Statistical Analysis

Statistical treatment was performed using IBM SPSS Statistics 26 software (IBM Corp., Armonk, NY, USA). The variables considered independent were ash, protein, fat, dietary fiber (total, soluble, and insoluble), caffeine, 3-, 4-, and 5-caffeoylquinic acid, 5-hydroxymethylfurfural, total phenolic and total flavonoid contents, as well as DPPH^•^ scavenging activity and ferric reducing antioxidant power. Pulp, husk, parchment, defective coffee beans, sieving residue, and silverskin were the dependent variables.

One-way ANOVA was used to reveal significant differences between samples, followed by Tukey′s post hoc test to make multiple comparisons. Statistical significance was taken as *p* < 0.05. Data were expressed as mean ± standard deviation of triplicate extractions.

## 3. Results and Discussion

### 3.1. Nutritional Composition of Coffee By-Products

Table 1 shows the nutritional composition of the coffee by-products. Except parchment, all of them seem to be good sources of protein. As described, coffee silverskin showed the highest content (16.3% dw), followed by the sieving residue (14.6% dw) and defective coffee beans (13.3% dw). However, it should be noted that in a previous study, Machado et al. [45] reported that about one quarter of total nitrogen of a coffee silverskin sample was non-protein nitrogen. By this way, the estimated amount through the Kjeldahl method could be slightly overestimated, and, in future studies, it would be important to analyze with the nitrogen profile of all these by-products in further detail. Moreover, it should also be highlighted that the presence of nitrogenous compounds in coffee by-products, such as caffeine, could also influence the total protein content determined by the Kjeldahl method [36]. Except for parchment, the nitrogen to protein conversion factors used in the present study were based on previous research where the specific factor for each by-product was calculated [36]. In general, the protein content estimated for silverskin and defective coffee beans are in agreement with those reported by Prandi et al. [36].

Coffee husk, a by-product of the dry processing method composed of skin, pulp, and parchment, presented a protein content (7.9% dw) significantly lower (*p* < 0.05) than that of coffee pulp (10.72% dw), a by-product of the wet method, composed only of skin and pulp. This is probably due to the presence of the parchment layer inside the coffee husk, which is very rich in total carbohydrates (specially in fiber), decreasing the contents of the other nutritional parameters per 100 g of sample. 

Overall, the results revealed that coffee by-products can be reasonable sources of protein with content comparable to other widely consumed foods such as quinoa (13.0%), buckwheat (14.8%), and millet (11.7%). Currently, finding economical and high nutritional quality plant protein sources as alternatives to animal sources is a challenge [50], and these by-products can be exploited in this context, but deeper knowledge of the nature of the protein such as its amino acid profile, digestibility, bioavailability, and anti-nutritional factors should be further explored.

According to the literature, coffee pulp is rich in minerals, particularly potassium, calcium, and magnesium [51] and, in fact, from all the samples herein analyzed, coffee pulp showed the highest content of ash (10.7% dw). However, this value was lower than that reported by Jiamjariyatam et al. [25] in Thai coffee pulp (16.7% dw). This difference may be attributed to the chemical composition and fertility of the soil in Thailand and Colombia, as these factors are highly correlated with the ash content of plants. Coffee silverskin also showed a very high value of total minerals (9.5%, Table 1), being potassium (∼5%), magnesium (2%), and calcium (0.6%) reported as its major macrominerals [52].

The highest fat content was observed in the defective coffee beans (8.5% dw), followed by the sieving residue (7.11% dw). Such high values were expected since the richness in total fat of regular green coffee beans (4–9%) is already known [53]. 

The remaining by-products were low in fat, with values ranging from 0.2 to 2.9% dw for parchment and silverskin, respectively. Previous data have shown that the main fatty acids present in coffee by-products are linoleic and palmitic acids [27,35,54].

Table 1 also shows that, compared with the other by-products, the parchment analyzed in this study is extremely rich in total dietary fiber (94.2% dw). Benitez et al. [40] reported a lower total dietary fiber value (77.2% dw) for parchment, and the differences between those results might remain in the different geographical origin of the samples, since our parchment came from Azores (in Portugal) and was characterized for the first time here. Azores have very specific edaphoclimatic conditions that could interfere in the chemical composition of the plants, and it would be interesting to explore this issue in future studies. This high content (94.2% dw) is of extreme relevance, since previous studies have shown that parchment fiber exhibits hypoglycemic and hypolipidemic properties [54]. In addition, Benitez et al. [39] also reported that parchment is mostly made up of xylans because xylose was the main monosaccharide. 

Regarding the other by-products, although lower, they also presented a high content of dietary fiber (39.0–65.9%), which was mostly insoluble one in all the by-products (Table 1). Notably, insoluble fiber is important in promoting bowel regularity, i.e., regular elimination of bulky/soft/easy-to-pass stools [55]. The soluble dietary fiber of the samples ranged from 0.6% dw, in parchment, to 9.1% dw, in coffee pulp. Based on the results obtained, coffee by-products show high potential to be used in the development of fiber-rich functional foods that help to control blood sugar levels and lower cholesterol levels. Moreover, according to the literature, coffee by-products can be metabolized by probiotic bacteria (*Lactobacillus* and *Bifidobacterium*) with production of beneficial metabolites (short-chain fatty acids) [56]. Thus, their inclusion in food products, such as bakery products, yogurts, or fermented milks, may provide a prebiotic effect.

### 3.2. Phenolic Compounds and Potential Antioxidant Activity of Coffee By-Products

The contents of total phenolics and flavonoids, as well as the antioxidant activity of the samples, are presented in Table 2. The defective coffee beans showed the highest TPC (6.54 g CGAE/100 g dw). This value was about 30% lower than that reported for non-defective green coffee beans (9.42 g CGAE/100 g dw) [57]. In a recent study with obese diabetic rats, Abdel-Mohsen et al. [58] observed that an aqueous extract of non-defective green coffee beans rich in phenolic compounds exhibited hepatoprotective activity against oxidative-stress-induced damage. Although defective coffee beans have a lower TFC than non-defective green coffee beans, it is still high and this by-product could be used to investigate hepatoprotective activity as well as other pharmacological activities. The sieving residue was the second by-product with the highest TPC (5.11 g CGAE/100 g dw), probably due to the presence of green coffee beans.

Coffee pulp and husk showed similar (*p* > 0.05) TPC (2.37 and 2.12 g CGAE/100 g dw, respectively). The TPC of the coffee husk was comparable to that found by Marques et al. (2.41 g CGAE/100 g dw) [59], higher than that found by Iriondo-DeHond et al. (1.56 g CGAE/100 g dw) [60], and lower than that found by Silva et al. (9.79 g CGAE/100 g dw) [28]. These variations in the TPC among the different studies can be due to several factors, such as the quantification method, extraction method, and geographical origin/species of the samples [28]. According to the literature, the main phenolic compounds found in aqueous extracts of coffee husks are chlorogenic acid, protocatechuic acid, kaempferol-3-O-galactoside, and gallic acid [30]. These bioactive compounds can play a crucial role in the prevention of chronic diseases like diabetes and obesity [30]. 

Silverskin and parchment are the coffee industry by-products with the lowest TPC, ranging from 1.28 to 0.18 g CGAE/100 g dw, respectively. The TPC of the silverskin herein studied falls within the range reported by Bessada et al. [35] for silverskin (*C. canephora*) from different geographical origins (0.52 to 2.03 g CGAE/100 g sample). This result was expected given that the silverskin under study is a combination of different coffee species and geographic origins. Furthermore, silverskin from *C. canephora* generally exhibits a lower TPC than silverskin from *C. arabica* [33], but these results could be due not only to the species but also to the roast degree of the samples, since higher roast degrees are usually employed to *C. canephora* beans. According to Benitez et al. [39], the incorporation of parchment in extruded snacks contributes to the antioxidant activity of the samples. When parchment flour is extruded, phenolic compounds are released, increasing its antioxidant activity [40]. Thus, temperature appears to improve the extraction of phenolic compounds from the parchment. In the present study, hydroethanolic extraction at room temperature was employed, so it is possible that the TPC of parchment was underestimated.

Coffee beans consist of a storage tissue where nutrients and phenolic compounds are formed and stored to support embryo development and plant reproduction [61]. Phenolic compounds contribute to plant survival by protecting the beans from biotic and abiotic stresses. Thus, it was expected that coffee beans would have a higher content of phenolic compounds including flavonoids compared to the layers surrounding the bean. However, the coffee pulp, husk, and silverskin had similar TFC (1.23, 0.88 and 0.70 g CE/100 g dw, respectively). The TFC of the silverskin is consistent with the results of McDonald et al. [32], who reported a TFC range of 0.19 to 0.86 g CE/100 g for silverskin of different varieties and roasting times. Moreover, the TFC of the coffee pulp, husk, and silverskin was comparable to flavonoid-rich foodstuffs such as apple pulp (0.71–1.00 g CE/100 g dw) and mulberry (1.02 g CE/100 g dw) [62,63]. With regard to the TFC of the parchment, it was higher than that found by Alkaltham et al. [41] (0.03 g CE/100 g dw, from Saudi Arabia), probably due to the geographical origin or variety of the samples. In turn, defective coffee beans and the sieving residue showed the highest TFC (5.22 and 4.91 g CE/100 g dw, respectively). Regarding the TFC of the defective coffee beans, it was higher than that found by Prandi et al. (1.62 g CE/100 g dw) [36].

Coffee by-products are rich sources of antioxidants, as determined by FRAP and DPPH^•^ inhibition assays. The DPPH^•^ results were considerably lower than the FRAP results, owing to the different principles and standards of each measurement method [64]. Due to the high TPC in defective coffee beans and sieving residue (Table 2), the FRAP (17.68 and 17.56 g FSE/100 g dw, respectively) and DPPH^•^ (3.22 and 2.85 g TE/100 g dw, respectively) assays also revealed a high antioxidant potential. The DPPH^•^ values (radical scavenging capacity) found for the defective beans are outside the range reported by Liao et al. [65] for acetonitrile/water extracts (1:1) of non-defective green coffee beans (7.63 to 9.20 g TE/100 g dw). This is probably due to the lower TPC found in our defective coffee beans compared to those non-defective coffee beans. 

In the FRAP assay, the coffee pulp exhibited nearly twice the reductive potential for ferric ions compared to coffee husk (8.58 and 4.57 g FSE/100 g dw, respectively). Although coffee pulp and husks have similar TPC, it is not possible to infer similar antioxidant activity for several reasons. Firstly, the TPC does not include all the antioxidants. Secondly, one must consider the synergic effects between the antioxidants present in the sample, which indicates that antioxidant activity is not only dependent on concentration [66]. Coffee pulp and husk are from different species and therefore may contain a different antioxidant profile. The result obtained for the pulp in this study was higher than that reported by Myo and Khat-Udomkiri (1.09 g FSE/100 g dried pulp) [26], where the extract was produced by ultrasonication with propylene glycol solvent (46.71%). However, the DPPH^•^ result of the pulp (0.77 g TE/100 g dw) was similar to that found by the same authors [26] (0.76 g TE/100 g dried pulp, *C. arabica* from Thailand) and Jiamjariyatam et al. [25] (0.84 g TE/100 g dw, *C. arabica* from Thailand). Some discrepancies may occur due to factors such as coffee variety, harvest location, and storage conditions, in addition to the extraction method. 

The FRAP value of silverskin is quite interesting. Silverskin has a significantly lower (*p* < 0.05, about 60%) TPC than coffee husk and exhibited a similar FRAP value to the coffee husk. Based on this, it seems that other antioxidant compounds besides phenolic compounds are involved in this activity. In fact, during the roasting of green coffee beans, phenolic compounds are linked to high molecular weight polymers known as melanoidins, which contain numerous hydroxyl groups and consequently high antioxidant activity [32,60]. These melanoidins produced by Maillard reactions are mostly composed of polysaccharides and can contribute to the antioxidant activity of silverskin [32,56].

The results of FRAP and DDPH^•^ tests show that hydroethanolic extracts of coffee by-products have antioxidant potential and can delay oxidative reactions through different mechanisms of action. Thus, coffee by-products may be of significance for extracting antioxidant compounds with possible application in the food industry. A recent study showed that adding a hydroethanolic husk extract to chicken burgers protects against lipid oxidation in a similar way to the synthetic antioxidant BHT [59].

### 3.3. Caffeine, Caffeoylquinic Acids, and 5-Hydroxymethylfurfural

Caffeine, 3-, 4-, 5-CQA, and HMF were quantified in all the by-products, and the results are shown in Table 3. 

Caffeine is an alkaloid compound with antibacterial and antifungal activity against various microorganisms [67]. Defective coffee beans can be considered the most caffeine-rich by-product of the coffee industry (1.40 g/100 g dw) with a value higher to that found in non-defective green coffee beans of the *Coffee arabica* species (0.44–0.50 g/100 g dw) [67]. However, this value can be justified due to the fact that the sample is composed of two species of coffee, *C. arabica* and *C. canephora*, the latter being richer in caffeine [68]. The sieving residue, pulp, and husk also showed significant amounts of caffeine. The caffeine content was significantly lower in the parchment (0.06 g/100 g dw), a value comparable to that reported by Mirón-Mérida et al. [42] (0.13 g/100 g sample). The results show that all these coffee by-products can be used as sources of caffeine.

In what concerns to the chlorogenic acids, the predominant isomer found in coffee by-products was 5-caffeoylquinic acid (5-CQA). The richest source of 5-CQA was defective coffee beans, followed by the sieving residue and coffee pulp (3.8, 2.5, and 0.2 g/100 g dw, respectively). Previously, Farah and Donangelo [69] estimated that the 5-CQA isomer accounts for 50 to 62% of total chlorogenic acids in non-defective green coffee beans. The 5-CQA in our defective coffee beans was similar to that found in non-defective green coffee beans of *Coffee arabica* (3.7 and 3.9 g/100 g, from Brazil and Kenya, respectively) [67]. Based on these results, it can be inferred that the extracts obtained from defective coffee beans and the sieving residue may exhibit potential to enhance human health owing to its high levels of CGA, particularly 5-CQA. Indeed, according to the literature, CGA-rich extracts obtained from non-defective green coffee beans were able to decrease metabolic syndrome markers in humans (blood pressure, lipid profile, glycemic control, insulin resistance, and appetite) [16]. Regarding coffee husk, the 5-CQA content (83.9 mg/100 g dw) was similar to that previously reported in the literature (84 mg/100 g dw) [28]. In addition, significant amounts of other caffeoylquinic acids (3-CQA and 4-CQA) were found, except for parchment. 

The chlorogenic acid and caffeine content of coffee by-products can also be influenced by various factors, such as species/variety/cultivar, degree of ripeness of the coffee cherry, agricultural methods, climate and soil conditions, post-harvest processing method, among others [42,67]. For example, Jiamjariyatam et al. [25] studied the effects of sun drying, hot air drying, vacuum drying, and freeze-drying on the TPC and CGA content in coffee pulp. The results showed that sun drying was less effective in preserving bioactive compounds due to the prolonged exposure to high temperatures or oxygen and continuous action of polyphenoloxidases.

During roasting, certain sugars are dehydrated, resulting in the formation of HMF. The HMF content of our silverskin sample was 39.5 mg/100 g dw, which was lower than the values reported by Bessada et al. [35] (283–321 mg/100 g silverskin), probably due to differences in the type of roast.

## 4. Conclusions

In this study, the chemical and bioactive composition of a wide range of by-products obtained during coffee cherry processing and coffee beans roasting is presented. The different coffee by-products have a distinct chemical composition, which broadens their potential applications. From all, the defective coffee beans and the sieving residue can be highlighted for their high antioxidant potential, which is associated with the high presence of phenolic compounds.

Extracting bioactive compounds from coffee by-products can be a strategy to increase the commercial added value of these residues. Especially, regarding caffeine and chlorogenic acids that are related to many nutraceutical activities such as antioxidant and anti-inflammatory activity. In addition, other compounds can be investigated in coffee by-products such as dietary fiber and protein.

Dietary fiber is, indeed, one of the main components of coffee by-products and can be exploited as a prebiotic ingredient. Consumers are increasingly considering gut health as a key to well-being, creating a market opportunity for coffee by-products. In future studies, investigating the nutritional quality of coffee by-product protein can also contribute to a new generation of protein sources with less environmental impact compared to animal sources.

Overall, all the coffee by-products studied here can be used as a source of sustainable functional ingredients contributing to health and well-being.

## Figures and Tables

**Table 1:** Nutritional composition of coffee by-products (% dw).

	Pulp	Husk	Parchment	Silverskin	Defective Beans	Sieving Residue
Ash	10.72 ± 0.21 ^a^	7.86 ± 0.07 ^c^	0.65 ± 0.05 ^f^	9.47 ± 0.06 ^b^	5.64 ± 0.04 ^e^	6.55 ± 0.08 ^d^
Protein	10.23 ± 0.08 ^d^	8.77 ± 0.14 ^e^	1.66 ± 0.07 ^f^	16.31 ± 0.12 ^a^	13.28 ± 0.07 ^c^	14.60 ± 0.08 ^b^
Fat	1.70 ± 0.01 ^d^	1.06 ± 0.07 ^e^	0.18 ± 0.03 ^f^	2.91 ± 0.09 ^c^	8.47 ± 0.22 ^a^	7.11 ± 0.37 ^b^
Total dietary fiber	46.12 ± 0.00 ^e^	39.04 ± 0.49 ^f^	94.19 ± 0.38 ^a^	65.87 ± 0.00 ^b^	57.00 ± 0.21 ^d^	60.67 ± 0.09 ^c^
Insoluble dietary fiber	36.99 ± 0.08 ^d^	32.13 ± 0.28 ^e^	93.62 ± 0.39 ^a^	56.86 ± 0.00 ^b^	56.08 ± 0.24 ^c^	56.19 ± 0.17 ^bc^
Soluble dietary fiber	9.13 ± 0.07 ^a^	6.91 ± 0.21 ^b^	0.57 ± 0.01 ^d^	9.01 ± 0.00 ^a^	0.93 ± 0.04 ^d^	4.48 ± 0.26 ^c^
Available carbohydrates	31.23 ± 0.29 ^b^	43.27 ± 0.48 ^a^	3.32 ± 0.28 ^f^	5.44 ± 0.24 ^e^	15.61 ± 0.36 ^c^	11.08 ± 0.26 ^d^

Results are the average of 3 independent experiments ± SD. Within each line, different superscript letters represent significant differences between samples (*p* < 0.05).

**Table 2:** Total phenolic compounds and in vitro antioxidant activity (DPPH• inhibition and FRAP assays) of coffee by-products (g/100 g dw).

	Pulp	Husks	Parchment	Silverskin	Defective Beans	Sieving Residue
TPC (CGAE)	2.37 ± 0.10 ^c^	2.12 ± 0.02 ^c^	0.18 ± 0.02 ^e^	1.28 ± 0.01 ^d^	6.54 ± 0.24 ^a^	5.11 ± 0.36 ^b^
TFC (CE)	1.23 ± 0.01 ^c^	0.88 ± 0.02 ^d^	0.08 ± 0.00 ^f^	0.70 ± 0.01 ^e^	5.23 ± 0.10 ^a^	4.91 ± 0.12 ^b^
FRAP (FSE)	8.58 ± 0.32 ^b^	4.57 ± 0.21 ^c^	0.35 ± 0.02 ^d^	4.05 ± 0.12 ^c^	17.68 ± 0.33 ^a^	17.56 ± 0.35 ^a^
DPPH^•^-SA (TE)	0.77 ± 0.10 ^b^	0.29 ± 0.11 ^c^	0.05 ± 0.00 ^d^	0.19 ± 0.05 ^c^	3.11 ± 0.03 ^a^	2.85 ± 0.18 ^a^

Results are the average of 3 independent experiments ± SD. In each line, different superscript letters represent significant differences between samples (*p* < 0.05). TPC, total phenolics content; CGAE, chlorogenic acid equivalents; TFC, total flavonoids content; CE, catechin equivalents; FRAP, ferric reducing antioxidant power; FSE, ferrous sulphate equivalents; DPPH^•^-SA, 2,2 diphenyl-1picrylhydrazyl radical scavenging activity; TE, Trolox equivalents.

**Table 3:** Caffeine (g/100 g), caffeoylquinic acids (mg/100 g), and 5-hydroxymethylfurfural (mg/100 g) content of coffee by-products.

	Pulp	Husk	Parchment	Silverskin	Defective Beans	Sieving Residue
Caffeine	0.85± 0.02 ^c^	0.46 ± 0.00 ^e^	0.06 ± 0.00 ^f^	0.71 ± 0.02 ^d^	1.40 ± 0.07 ^a^	1.12 ± 0.01 ^b^
3-CQA	6.54 ± 0.14 ^c^	4.01 ± 0.12 ^c^	n.d.	9.44 ± 0.22 ^c^	408.20 ± 22.63 ^a^	323.10 ± 5.42 ^b^
5-CQA	220.56 ± 6.99 ^c^	83.93 ± 1.09 ^cd^	5.36 ± 0.57 ^d^	52.53 ± 0.83 ^cd^	3787.58 ± 147.35 ^a^	2533.06 ± 44.76 ^b^
4-CQA	14.83 ± 1.05 ^c^	11.82 ± 0.05 ^c^	n.d.	17.71 ± 0.30 ^c^	684.96 ± 28.31 ^a^	484.65 ± 6.34 ^b^
HMF	-	-	-	39.52 ± 1.07	-	-

Results are the average of 3 independent experiments ± SD. In each line, different superscript letters represent significant differences between samples (*p* < 0.05). CQA, caffeoylquinic acid; HMF, 5-hydroxymethylfurfural; n.d., non-detected.

## Data Availability

The data are included in the article.
